# Who Cares about Forests and Why? Individual Values Attributed to Forests in a Post-Frontier Region in Amazonia

**DOI:** 10.1371/journal.pone.0167691

**Published:** 2016-12-12

**Authors:** Patricia Carignano Torres, Carla Morsello, Luke Parry, Renata Pardini

**Affiliations:** 1 Departamento de Ecologia, Instituto de Biociências, Universidade de São Paulo, Cidade Universitária, São Paulo, São Paulo, Brazil; 2 Escola de Artes, Ciências e Humanidades, Universidade de São Paulo, Ermelino Matarazzo, São Paulo, São Paulo, Brazil; 3 Lancaster Environment Centre, Lancaster University, Lancaster, Lancashire, United Kingdom; 4 Núcleo de Altos Estudos Amazônicos (NAEA), Universidade Federal do Pará, Belém, Pará, Brazil; 5 Departamento de Zoologia, Instituto de Biociências, Universidade de São Paulo, Cidade Universitária, São Paulo, São Paulo, Brazil; Chinese Academy of Forestry, CHINA

## Abstract

Understanding the multiple ways people value forests is important, as individual values regarding nature have been shown to partly determine willingness to participate in conservation initiatives. As individual values are influenced by past experiences, the way people value forests may be related to the ecosystem services they use and receive. We here aim to investigate if people value forests because of material and non-material benefits forest provide (material and non-material values), and if these values are defined by previous experiences associated with using forest resources and having frequent contact with forests. By interviewing 363 residents across 20 landscapes varying in forest cover in a post-frontier region in Amazonia, we evaluated: (1) if the use of forest resources—especially bushmeat, important for sustenance and cash income in virtually all tropical forests—is associated with attributing higher material value to forests; (2) whether the contact with forest (estimated by local forest cover and visits to forests) is associated with attributing higher non-material value to forests. As expected, respondents from households where hunting occurs and bushmeat consumption is more frequent attributed higher material value to forests, and those living in more deforested landscapes and that visited forests less often attributed lower non-material value to forests. The importance of bushmeat in shaping the way people value forests suggests that encouraging the sustainable use of this product will encourage forest conservation. Results also point to a potential dangerous reinforcing cycle: low forest cover and the loss of contact with forests may erode forest values and facilitate further deforestation. Engaging rural communities in forest conservation initiatives is challenging yet urgent in degraded landscapes, although harnessing appreciation for bushmeat could offer a starting point.

## Introduction

Individual human values (concepts and beliefs about desirable end states or behaviors; [1]) are fundamental for understanding people’s attitudes and behavior [2, 3, 4, 5]. In particular, values people attribute to nature and the environment are associated with pro-environmental attitudes and behaviors [5, 6, 7, 8]. These linkages are receiving increasing attention in conservation, with the recognition that understanding how and why (and to what extent) people value non-human nature (e.g. species, ecosystems) can be a useful tool for environmental management and biodiversity conservation [9, 10, 11]. Understanding diversity in those values is important for anticipating and resolving conflicts among stakeholders, promoting socially-acceptable management practices and engaging people in conservation by considering people’s natural resource use and what they prioritize [12, 13, 14, 15, 16]. Importantly, individual values, while influencing attitude and behavior, can themselves be influenced by life experiences, such as past behavior [17, 18]. In particular, prior experiences with nature may affect the way people value it [19, 20]. For formulating effective conservation strategies, it is thus important to understand which experiences determine individual values towards nature because these values are expected to be associated with pro-environmental behavior.

The ecosystem services approach, in which maintaining the benefits we derive from nature is taken as the starting point for justifying the conservation of ecosystems [21], is increasingly been advocated for, and used in, conservation strategies [22, 23, 24]. However, despite the fact that humans may value non-human nature in multiple ways [5, 15], few studies have investigated if people value nature because of the benefits and services ecosystems provide [16].

These benefits and services are diverse, including not only material benefits, such as the provision of resources as food, water or fuel, but also non-material benefits, such as recreation, cultural or spiritual well-being, and the satisfaction of knowing a species or habitat exists or that future generations may benefit from nature [21, 25, 26, 27]. Nevertheless, the incorporation of ecosystem services into conservation strategies has focused primarily on economic incentives, and thus on the benefits that can be more easily monetized, such as many of the provisioning services (e.g. timber, food) or public goods (fresh water supply) associated with material benefits [27]. However, ecosystems provide a broader set of services [25, 26], such as cultural services [27] related to spiritual enrichment and aesthetic experiences [21], which are associated with non-material benefits [27]. There is growing awareness that not only material but also non-material benefits may influence individual values towards nature, and thus people’s pro-environmental behavior [28], such as maintaining a forest patch or the voluntary participation in community harvest agreements.

Previous experience in using forest resources may then influence individual values, with people that use forest resources attributing value to forests because they provide material benefits (called here as material value). Bushmeat provision, in particular, is a very important and widespread ecosystem service provided by forests in developing countries, supplying both sustenance and cash income [29, 30]. Although the importance of bushmeat for livelihoods in tropical forest regions is well-established [29], the association between bushmeat (hunting and consumption) and how people value forests has received scant attention (but see [31]). This is a research gap, considering that conserving tropical forests depends partly on voluntary participation by forest and rural dwellers (e.g. in the establishment of sustainable use reserves or community harvest agreements or rule enforcement) and that bushmeat is a key ecosystem service that tropical forests provide to local people. While overhunting has been associated with decline and even extinction of game species [32], the sustainable use of this forest resource may in fact increase the value attributed to forests by those living within and nearby forests thus enhancing their willingness to conserve them.

On the other hand, people attributing non-material values to forests (i.e. attributing value to forests because they provide aesthetic, spiritual or cultural benefits) may result from the previous contact people have had with forests. The disconnection and loss of direct contact with nature can lead to less favorable perceptions about nature and its biodiversity, resulting in negative attitudes to biodiversity conservation [19, 20]. Whilst the erosion of the value attributed to nature has been shown to follow urbanization [19, 33], it is unclear whether the more subtle process of cumulative deforestation in rural areas reduces the non-material value attributed to forests and, thus reduces the willingness to conserve forest remnants. As in rural areas direct contact with forests are frequently associated with hunting or other activities that imply visiting and knowing forests, a decline in these forest activities may also contribute to the erosion of non-material value of forests.

We here aim to investigate how and why rural people value forests. In particular, if they value forests because of material and non-material benefits forest provide (material and non-material values), and if these individual values are defined by previous experiences associated with hunting and consuming bushmeat (a very important forest resource), and having frequent contact with forests (either by living in forested areas or making frequent visits to the forest). We then quantified material and non-material values people attribute to forests across a heterogeneous post-frontier region in eastern Amazonia. Post-frontier regions usually encompasses spatial gradients in remaining forest cover [34] and therefore spatial heterogeneity in the availability and use of bushmeat [35, 36, 37] as well as in the potential human contact with forests. These regions are thus well-suited to investigate the association of deforestation, visits to forests and bushmeat harvest and consumption with the values people attribute to forest. We conducted an interview-based survey [38] with 363 rural residents living in 20 landscapes with varying forest cover, to quantify individual values as well as the use of forest resources and frequency of forest-based activities. We hypothesized that hunting and consumption of bushmeat should be associated with the material value people attribute to forests, while local forest cover and more frequent visits to forests should be associated with the non-material value people attribute to forests.

## Materials and Methods

### Study region

We conducted this study in the eastern Brazilian Amazon, south of the city of Santarém, in Pará state. Although inhabited since pre-Colombian times by autochthonous populations, the city was officially founded in 1661 and since then has received successive waves of migrants associated with economic cycles (e.g., sugar cane, cocoa, rubber, timber [39, 40]), which transformed the municipality into the main commercial center of the Lower Amazon [41]. More recent major in-migration started around 1958, with road construction, government-led colonization and land reform initiatives that promoted the influx of low-income migrants from the arid Northeast and the South of Brazil [42, 43]. Agriculture mechanization began at the end of the 1990s, with a new wave of in-migration of farmers from the South and Mid-west of Brazil [43]. The area is therefore home to both recent and long-term in-migrants from various regions of Brazil.

**Fig 1:**
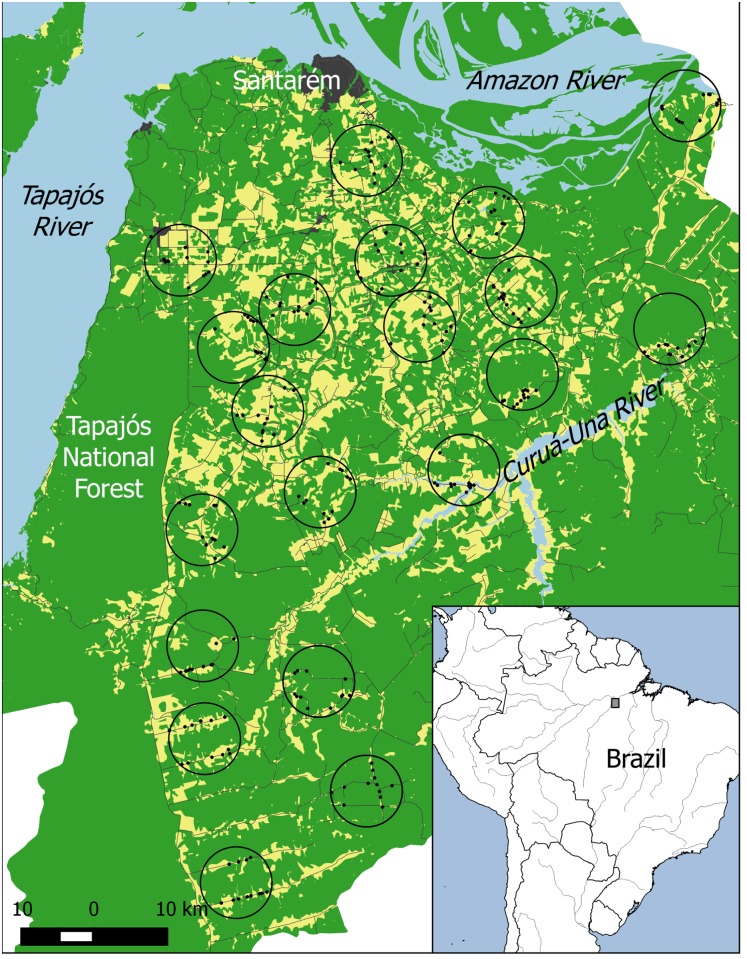
Land cover map of the study region. Location of the study region in South America and land-cover map of the study region, indicating the location of the 20 study landscapes (black circumferences) and the 240 sampled households (black dots). Urban areas in dark grey, forests in green, converted land in yellow, water bodies in blue and roads in grey.

The study region from where we drew our sample was c. 1 million ha, bordered by the Rivers Amazon, Tapajós and Curuá-Una. More than half of the area is covered by primary or secondary forest, with larger tracts of continuous primary forest mostly further from urban centers (Fig 1). The study area encompasses the rural and peri-urban areas of the municipalities of Santarém, Belterra and Mojuí dos Campos, with a total population of 310,898 inhabitants, of which c. 85,000 live in rural settings [44].

### Sampling design

We used a hierarchical sampling design, first selecting landscapes within the study region that captured the variability in forest cover, and then selecting households within each landscape to interview household heads. In total, we sampled 20 7,850-ha landscapes (each had a 5-km radius), distributed across a gradient of current forest cover from 33 to 93% (Fig 1). The 20 sampled landscapes encompassed 70 rural communities, varying from 10 to 400 households.

Within each of the 20 landscapes, we randomly selected 12 households. After mapping roads, rivers and households within each landscape in the field with the help of Google Earth images, we used ArcGis 9.3 to establish a set of XY locations every 10 m along all inhabited roads and rivers. We then randomly selected 12 points per landscape at least 400 m apart from each other (the maximum possible distance in all landscapes) and targeted the nearest households to each selected location. If a same household was the nearest to more than one of these points, the second point was excluded and another one was randomly drawn. If a selected point was equidistant to more than one household, we randomly selected one of these households. We skipped individual households if the household head(s) declined to take part in the study (n = 3), or if after three visits no resident was encountered (n = 4), sampling the next nearest household.

In total, we selected 240 households and interviewed both household heads (man and woman) in most of them. Household heads were interviewed simultaneously, but separately, each by one interviewer. When one of the household heads was absent, we revisited the household on another occasion and conducted the interview with only one of the heads if the other was again absent. In 77 of the 240 households, we could not interview both household heads (in 29 we could not interview the woman and in 48 the man). In total, we interviewed 363 residents, 190 women and 173 men.

### Forest values and their determinants

We used an interview-based survey (i.e. structured questionnaire) with household heads, applied by PCT and a team of three trained assistants, who conducted the interviews between July and December 2013. Using the interview-based survey, we quantified: (i) material and non-material values people attribute to forests using a Likert-type scale; (ii) independent variables, i.e. hunting and consumption of bushmeat, and the frequency of visits to the forest; and (iii) control variables. Before data gathering, the survey was pre-tested in the field for comprehension and adjusted accordingly.

#### Forest values

We measured two types of individual values people can attribute to forest because of the benefits forests can provide: material and non-material values [27]. For each of the two types of values, we constructed a Likert-type scale (hereafter Likert scale) composed of six statements (items), each statement being a single sentence expressing a point of view, a belief or an emotional feeling [45] favorable or unfavorable in relation to forests, for which the respondent was asked to agree or disagree. Because an appropriate constructed scale should have an equal proportion of positive (favorable) and negative (unfavorable) statements [45], for each scale we included three positive and three negative statements. All statements were in Portuguese, using language adequate to respondents with low levels of schooling or illiterates. The statements regarding the material value focused on valuing forests for their potential for providing or disrupting provisioning services (material benefits or losses), such as (i) cash income, (ii) the extraction of products for food or other purposes, and (iii) production losses or reduction of other income opportunities. The statements regarding the non-material value of forests focused on valuing forests for their potential for providing aesthetic, spiritual or cultural well-being and/or satisfaction in knowing forests exist now and for future generations (non-material benefits): (i) enjoying experiences arising from direct contact with forests, (ii) considering forest important to future generations, (iii) caring for forest animals, and (iv) fearing wildlife close to home. People’s reply to each statement was in a Likert-type response format, i.e. “strongly agree”, “agree”, “neutral/or could not answer”, “disagree”, and “strongly disagree”. To each answer, we assigned points from 1 (very unfavorable) to 5 (very favorable), with 3 as “neutral/or could not answer” (undecided position [46]). For each respondent and type of value, we calculated a score summing up the points from each of the six statements (range = 6 to 30), with higher scores reflecting a higher value attributed to forests.

#### Predictors of forest values

We considered hunting and bushmeat consumption as predictors of the material value of forests, and forest cover and visits to forests (whether for hunting or other forest activities) as predictors of the non-material value of forests. Because there are several possible manners to quantify each of these predictors, we quantified different measures or proxies for each of them and selected the one that best explained the variation in the Likert scale for either material or non-material values using a model selection approach (S1 Text).

We calculated bushmeat consumption with three different proxies, for all of which we considered the consumption in the household during the 15 days prior to the interview: the total number of meals that contained bushmeat (considering lunch and dinner), the total quantity of consumed bushmeat (in kilograms), and the quantity of consumed bushmeat per adult equivalent (in kilograms). To calculate adult equivalent per household, children below 15 years and adults above 65 years were assigned a weight of 0.5, while all other household members (15–65 years) were assigned a weight of 1 [47]. The total number of meals that contained bushmeat, varying from 0 to 14 meals (mean = 0.8, SD = 1.8), was the proxy that best explained the variation in the material value attributed to forests (S1 Table).

We calculated four alternative proxies of hunting always considering the activities of all household members during the 30 days prior to the interview: whether any household member had gone hunting, whether any household member had gone hunting and succeeded, the total number of hunting events, and the total hunting offtake (total kilograms of hunted animals). Whether any household member had gone hunting was the proxy that best explained the variation in the material value attributed to forests (S1 Table). A member had gone hunting in 30% of the sampled households.

We measured the amount of forest cover (in km^2^) at five different spatial scales around each interviewee residence: 1-km radius buffer (3.1 km^2^), 2-km (12.6 km^2^), 3-km (28.3 km^2^), 4-km (50.2 km^2^) and 5-km (78.5 km^2^). For each spatial scale, forest cover was quantified considering either: only non-degraded primary forest, total primary forest (degraded and non-degraded), or total forest (total primary plus secondary forest older than 10 years). Total forest (total primary plus secondary forest older than 10 years) at 1-km radius buffer, which varied from 0.1 to 2.8 km^2^ (mean = 1, SD = 0.7), was the proxy that best explained the variation in the non-material value attributed to forests (S2 Table).

Visits to forests refer to those specifically for hunting, or for other activities, such as the extraction of other forest products, opening new agricultural fields, surveilling the property or simply crossing the forest to get somewhere else. In this case, we calculated seven proxies to quantify respondents’ visits to forests instead of considering all household members (as we wanted to estimate individual direct contact with forest). For all proxies we considered the 15 days prior to the interview: whether the respondent had gone hunting in the forest, whether the respondent had visited the forest (regardless of the type of activity), the total number of hunting events in the forest, the total number of visits to the forest (regardless of the type of activity), the total time spent hunting in the forest (in minutes), and the total time spent in the forest (regardless of the type of activity) (in minutes). Whether the respondent had visited the forest in the last 15 days was the proxy that best explained the variation in the non-material value attributed to forests (S2 Table). Twenty-six percent of the respondents reported having visited the forest (18% of the interviewed women and 48% of the interviewed men).

#### Control variables

To control for confounding factors that could also influence both values attributed to forests, three variables were considered: gender, whether the respondent was born in the community of residence or not (origin), and an estimate of household-level dependency on forest products other than bushmeat (as we already consider the importance of bushmeat as a dependent variable), all of which had been previously associated with the value people attribute to protected areas and with conservation attitudes [31, 48, 49, 50, 51]. While gender and origin were incorporated in the analyses of both types of values attributed to forests, dependency on forest products other than bushmeat was included only in the analyses for the material value attributed to forests to account for all other forest products that people could extract from forests other than bushmeat.

We estimated dependency on forest products other than bushmeat as the proportion of the total household income in the last 30 days [52] originating from the extraction of any forest products except bushmeat. To calculate total income we summed the monetary income and the non-monetary income from products produced or collected for subsistence. The latter was estimated by the commercial values of the products declared by each respondent (own-reported values), the mean commercial value of the products declared by other respondents living in the same or in the closest landscape (when the respondent was unable to inform the commercial value), or the commercial value of the closest substitutes (when no one was able to attribute a commercial value to the good, as frequently occurs with untraded forest products [53]).

Nineteen percent out of the 173 male and 20 percent of the 190 female interviewees were born in their community of residence. Sixty one percent of the respondents reported the extraction of forest products other than bushmeat in the household during the last month, with dependency on these products varying from 0 to 99% (mean = 6, SD = 13).

Correlations between the predictors and control variables were generally weak (r < 0.30) for both types of value attributed to forests. The only value larger than this was between bushmeat consumption and hunting (r = 0.44).

### Data analysis

#### Scale evaluation

We evaluated our Likert scales in terms of their unidimensionality and internal consistency (reliability). Unidimensionality indicates weather the scale encompasses only one concept or construct [45]. Internal consistency is a measure of item-total correlations and thus reliability of the scale, therefore describing the extent to which all items in a test measure the same concept or construct [54, 55]. To evaluate the unidimensionality of each of the two Likert scales (one for each value type), we conducted a principal component analysis (PCA). To test the internal consistency and thus reliability of each scale, we adopted the Cronbach’s α [56, 57] (S2 Text). All these exploratory analyses were implemented in R 3.0.3 [58], using the *psych* package [59].

From the PCAs, we found that in both cases the first component was much more important in explaining the variation in the dataset (S3 Table), with the eigenvalue of the second component only slightly higher than 1, therefore unimportant from a variance perspective [60]. As expected when creating a proper scale, the items were correlated (S1 Fig). The Cronbach’s α among the 6 items of each scale was lower than the typical cut-off value (> 0.70), α = 0.64 for the material value attributed to forests and α = 0.56 for the non-material value attributed to forests. Yet the values are reasonable [61, 62], as α is strongly influenced by the number of items in the scale [56]. Thus the values we obtained are associated with the relatively low number of items (6) in the scales, and do not indicate lack of reliability [56]. Although the unidimensionality of the scales improved when one item from the material value scale and two items from the non-material value scale were excluded (S4 Table, S2 Fig), the Cronbach’s α values did not change for the material value scale and slightly decreased for the non-material value (α = 0.55). We then decided to analyze the data for the 6-items scales, since a higher number of items increases the linearity of the scale [57] and because the number of dimensions are often sample specific and, for this reason, some researchers see unidimensionality as a rather unrealistic goal [54].

#### Predictors of forest values

We investigated the relationship between individual values attributed to forest and the predictor and control variables, considering each respondent as the sampling unit and his/hers scores in each of the two Likert scales as the dependent variables, using a model selection approach. We used generalized linear mixed-effects models (GLMM) to account for the hierarchical nature of the sampling design, and thus for the non-independence of data coming from the same landscape and household [63]. We modeled the dependent variables with a normal distribution and used linear function (link identity), because Likert scales, created by summing up the values of several items, are not considered ordinal scales but rather produce interval data [57]. All continuous independent variables (forest cover, bushmeat consumption and forest dependency) were standardized so that each had a mean of zero and a standard deviation of one, a procedure adopted that helps to improve convergence of the fitting algorithm [63].

For each type of value attributed to forests, we compared a set of candidate models: an intercept-only model for reference (with no fixed factors, therefore no independent variables), simple models with each of the independent variables (either predictors or control variables) on their own as fixed factors, and models with all possible combinations among independent variables as fixed factors, without accounting for interactions between them. In all models, landscape was considered a random factor to account for the non-independence of multiple observations within the same landscape. For the material value attributed to forests, models also included household as a random factor to take into account non-independence within the same household (both household heads were interviewed in most households). As we estimated visits to forests only for the household head that more often performed forest activities, all respondents included in the analyses of the non-material value attributed to forest were from distinct households. Hence, household was not included as a random factor in this case, and the number of respondents was lower (239 respondents instead of 363).

Alternative models were compared using the difference in their AICc values (second-order Akaike’s Information Criterion, for small sample size) in relation to the first-ranked model (ΔAICc) [64]. Akaike’s Information Criterion is a measure of the fit of a model to the data that takes into account the number of model parameters, and the smaller the AIC, the better the fit [65]. We adopted a value of ΔAICc ≤ 2 to indicate equally plausible models. All analyses were implemented in R 3.0.3 [58] using the lme4 package [66].

### Ethics statement

Our study protocol was evaluated and approved by a Research Ethics Committee from the Brazilian National Commission for Research Ethics (CAAE 16766413.4.0000.5464 in Plataforma Brasil). Prior to the interviews, we contacted all representatives of the riverine and rural communities where our study took place, explained the research and obtained their written voluntary and informed consent. We then obtained written voluntary and informed consent from each participant prior to the beginning of the interview. They were explained about the research aims and that their participation was voluntary, that they could withdraw at any time and that information would be used anonymously.

## Results

**Fig 2:**
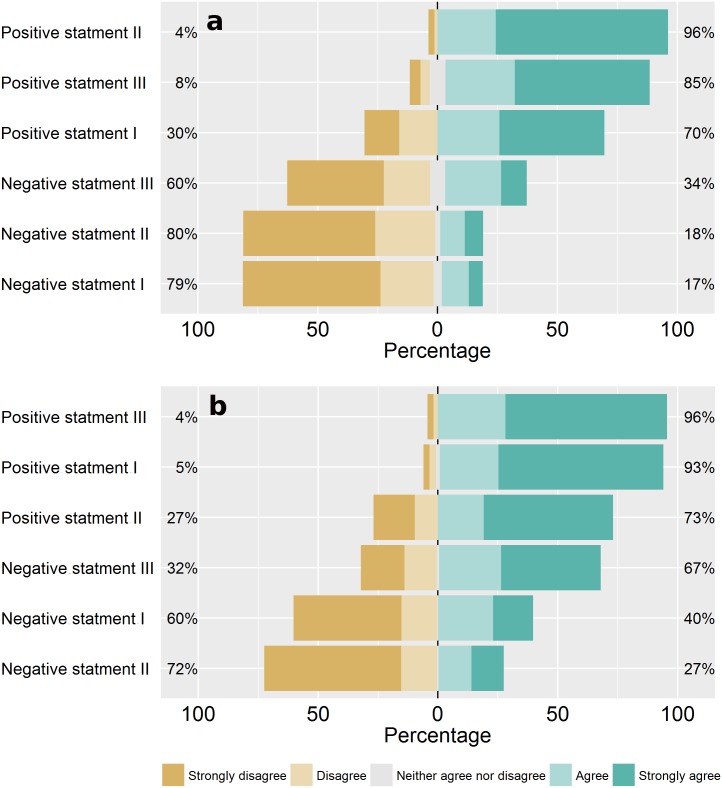
Responses to the statements used to quantify individual values attributed to forests. Percentage of responses, in a 5-item Likert type scale, to each of the six statement used for (a) the material value and (b) the non-material value attributed to forests.

**Fig 3:**
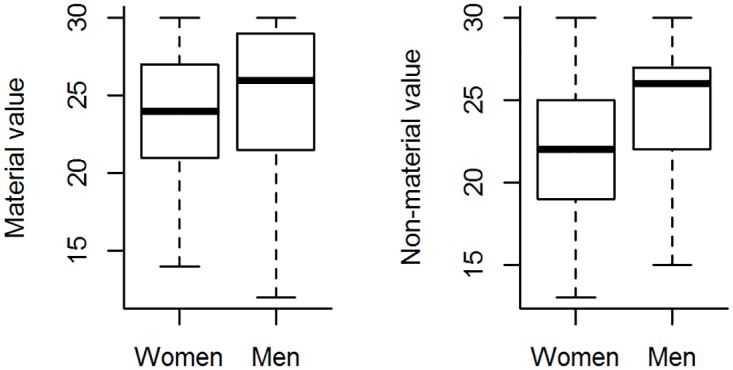
Variation in individual values attributed to forests between men and women among residents in the study region. (a) Material and (b) non-material values attributed to forests, as Likert scale scores. Boxplot showing the median (horizontal line), 1^st^ quartile (lower whisker), 2^nd^ quartile (lower box), 3^rd^ quartile (upper box) and 4^th^ quartile (upper whisker).

Respondents generally agreed with the favorable (and disagreed with the unfavorable) statements on forests (Fig 2), attributing high values to forests (i.e. high values on the Likert scale), which indicates that most people see forests as important (material value—range 7 to 30, mean = 24.3, SD = 4.5; non-material value—range 10 to 30, mean = 23.8, SD = 4.5). However, men valued forests more than women did, particularly when considering the non-material value (Fig 3).

### Predictors of forest values

As expected, bushmeat consumption and hunting were positively associated with the material value people attributed to forest (i.e. these variables were present with positive coefficients in two or three of the selected models, respectively; Table 1). People from households where bushmeat consumption was more frequent (number of meals containing bushmeat in the last 15 days) and hunting occurs (whether any household member had gone hunting in the last 30 days) attributed higher material value to forests (Table 1). However, selected models with the variables bushmeat consumption and hunting also included origin or both origin and gender. Hunting was included in three of the five selected models, including the top model, whereas bushmeat consumption was included in two (one of them that also included hunting). All five selected models contained origin and four of them both origin and gender, indicating that respondents that were born in the community of residence and men attributed higher material value to forests (Table 1).

**Table 1:** Model selection results for the predictors of the material value attributed to forests among 363 rural residents. All candidate models are GLMM models with landscapes and households as a random factor. Only candidate models with weight ≥ 0.02 are shown.

Model description	K	logLik	AICc	ΔAICc	ωi	Origin	Gender	Hunting	Bushmeat Consumption	Forest Dependency
**Origin+gender+hunting**	**7**	**-1055.8**	**2126.0**	**0.0**	**0.18**	**1.17 (0.59)**	**0.85 (0.45)**	**0.87 (0.53)**		
**Origin+gender**	**6**	**-1057.5**	**2127.2**	**1.2**	**0.10**	**1.26 (0.59)**	**0.85 (0.45)**			
**Origin+gender+consumption**	**7**	**-1056.6**	**2127.5**	**1.5**	**0.09**	**1.29 (0.59)**	**0.86 (0.45)**		**0.4 (0.24)**	
**Origin+hunting**	**6**	**-1057.7**	**2127.6**	**1.6**	**0.08**	**1.17 (0.59)**		**0.87 (0.53)**		
**Origin+gender+hunting+consumption**	**8**	**-1055.7**	**2127.7**	**1.7**	**0.08**	**1.21 (0.59)**	**0.85 (0.45)**	***0*.*61 (0*.*58)***	***0*.*29 (0*.*26)***	
Gender+hunting	6	-1058.2	2128.6	2.6	0.05		0.85 (0.45)	0.97 (0.53)		
Origin	5	-1059.3	2128.8	2.8	0.05	1.26 (0.59)				
Origin+gender+hunting+forest dependency	8	-1056.2	2128.9	2.9	0.04	1.16 (0.59)	0.85 (0.45)	0.83 (0.54)		*0*.*10 (0*.*25)*
Origin+consumption	6	-1058.5	2129.2	3.2	0.04	1.29 (0.59)			0.39 (0.24)	
Origin+hunting+consumption	7	-1057.5	2129.4	3.4	0.03	1.21 (0.59)		*0*.*61 (0*.*58)*	*0*.*38 (0*.*26)*	
Origin+gender+forest dependency	7	-1057.7	2129.8	3.8	0.03	1.25 (0.59)	0.85 (0.46)			*0*.*16 (0*.*24)*
Hunting	5	-1060.0	2130.2	4.2	0.02			0.97 (0.53)		
Origin+gender+consumption+forest dependency	8	-1057.0	2130.3	4.3	0.02	1.28 (0.59)	0.86 (0.45)		0.39 (0.24)	*0*.*13 (0*.*24)*
Gender	5	-1060.1	2130.4	4.4	0.02		0.85 (0.46)			

Selected models (ΔAICc<2) in bold; K: number of parameters; logLik: log-Likelihood of the model; AICc: AICc value; ΔAICc: difference in AICc value compared to the first-ranked model; ωi: Akaike weight (the relative likelihood of the model, given the data, interpreted as probabilities); coefficients for each variable of the model. Coefficient in italic indicates that its 85% confidence interval includes zero [67].

As expected, forest visits and forest cover were positively associated with the non-material value people attributed to forest (i.e. these variables were present with positive coefficients in all three, or the two first-ranked, selected models, respectively; Table 2). People that had visited the forest in the last 15 days and that live in more forested landscapes (non-degraded primary forest at 1-km radius buffer) attributed higher non-material value to forests than those who visited the forest less often and live in less forested landscapes. Gender was as important as having visited the forest, being also present in all three selected models. Again, men attributed higher non-material value to forests than women did. Origin was also included in one of three selected models; however, the confidence interval for the coefficient included zero (thus not necessarily people born in the community of residence attributed higher non-material value to forests; Table 2).

**Table 2:** Model selection results for the predictors of the non-material value attributed to forests among 239 rural residents. All candidate models are GLMM models with landscapes as a random factor. Only candidate models with weight ≥ 0.02 are shown.

Model description	K	logLik	AICc	ΔAICc	ωi	Gender	Contact with forests	Forest cover	Origin
**Gender+visits to forests+forest cover**	6	-683.0	1378.4	0.0	0.34	**2.05 (0.64)**	**1.45 (0.60)**	**0.66 (0.30)**	
**Gender+visits to forests+forest cover+origin**	7	-682.1	1378.6	0.2	0.31	**2.09 (0.64)**	**1.40 (0.60)**	**0.70 (0.30)**	*0*.*63 (0*.*69)*
**Gender+visits to forests**	5	-684.9	1380.2	1.7	0.14	**2.16 (0.64)**	**1.75 (0.60)**		
Gender+contact with forests+origin	6	-684.2	1380.8	2.4	0.11	2.20 (0.64)	1.72 (0.59)		*0*.*42 (0*.*69)*
Gender+forest cover+origin	6	-685.1	1382.5	4.1	0.04	2.42 (0.62)		0.87 (0.29)	*0*.*80 (0*.*69)*
Gender+forest cover	5	-686.3	1382.9	4.4	0.04	2.39 (0.62)		0.82 (0.28)	

Selected models (ΔAICc<2) in bold; K: number of parameters; logLik: log-Likelihood of the model; AICc: AICc value; ΔAICc: difference in AICc value compared to the first-ranked model; ωi: Akaike weight (the relative likelihood of the model, given the data, interpreted as probabilities); coefficients for each variable of the model. Coefficient in italic indicates that its 85% confidence interval includes zero [67].

## Discussion

Our results strongly suggest that both hunting and bushmeat consumption by rural Amazonians is linked, as predicted, with higher material value attributed to forests, irrespectively from how dependent people are on forest resources other than bushmeat. Furthermore, people living in more forested landscapes—and especially those visiting forests more frequently—attributed higher non-material value to forests. Those born in their community of residence perceived forests to have higher material value, and men perceived forests to have both higher material and non-material values. We discuss these results in the following paragraphs, and go on to consider their significance for conservation initiatives.

Our findings indicate that both hunting and bushmeat consumption are likely to be important in maintaining the value people attribute to forests. To our knowledge, this is the first study to examine, at the household level, this association between hunting and bushmeat consumption and the value people attribute to forests. This builds on recent evidence that village-scale hunting activity is positively associated with economic value attributed to forests by individual residents in Borneo [31]. Likewise, the availability of wild animals for food increases the value attributed to forests by farmers living nearby forest reserves in Ghana, given their importance for immediate subsistence and people’s livelihoods [68]. These two studies, however, do not distinguish between bushmeat harvest and consumption, which are not necessarily correlated, and do not control for the dependency on other forest resources. Bushmeat consumption in Amazonia is typically more widespread than hunting [37, 69] because trading and sharing are important means of acquiring bushmeat [30]. Our results suggest that bushmeat is a valued resource that influence the perceived value of forests, even among consumers that did not hunt, enhancing the importance of forests and game species to a wider number of households. Moreover, we show that both bushmeat consumption and hunting seem to provide a strong reason to value forests, irrespective of how dependent people are on forest products other than bushmeat.

We also found that whether any household member had gone hunting was a better predictor of the value attributed to forests than continuous variables, such as the number of hunting events or total offtake. This may be due to the fact that hunting events were reported only in 30% of households in the study region, making a binary variable the better option where hunting is not widespread across households. For consumption, the number of meals that contained bushmeat was the best proxy, rather than either the total quantity consumed in the household or the quantity consumed by adult equivalent, variables commonly quantified among previous studies (e.g. [30, 36, 70, 71, 72, 73]). This may suggest that the frequency with which household members consume bushmeat, regardless the weight consumed, is important to determine how people value forests.

Our results also highlight the major importance of people’s contact with forests, provided by activities that require visiting forest locations, such as hunting and the extraction of other forest products, or by simply having to cross a forest tract, as a predictor of the non-material value attributed to forests. The loss of direct contact, caused by a decrease in outdoor activities, has also been linked to the erosion of the value attributed to nature by children [20]. Interestingly, though, our results show that irrespective of respondents visiting the forest, reduction in landscape forest cover caused by deforestation is also associated with a lower non-material value attributed to forests. The measure of forest cover that best explained the non-material value was at the smallest spatial scale (i.e. within 1 km from the household), indicating that close proximity to forests is important. Indeed the ‘extinction’ of experiences with natural environments, a consequence of urbanization, is posited as the main driver of humans ‘de-valuing’ nature [19]. Our results are thus congruent with the idea that the erosion of values attributed to non-human nature may also occur in rural settings through the processes of habitat loss.

Our findings corroborates that gender is a strong predictor of both material and non-material values attributed to forests, whereas origin (in our case, being born in the community of residence) is an important predictor of the material value only. That men value forests for what they ‘provide’ (relatively more than women do) has previously been reported for people leaving near protected areas or forests [14, 74] as well as for the general public in developed countries [75]. This male-preference has been reported also for cultural and spiritual values attributed to forests, and it was suggested this was because men visit forests more frequently than women do and spend more time engaged in forest-related activities such as extraction of forest resources for food and fuel [14]. Indeed forest-related activities in our study region are also usually performed by men. Origin, which in our case is correlated with long residence in the community, may be linked to traditional ecological knowledge [76] and thus we would expect adults locally born to be more aware of forest utility than those born elsewhere are. Length of residence in a village has been linked to perceptions of higher economic importance attributed to forests [31], although the reasons for this are unclear. In any case, our findings suggest that origin, or long residence in the community, is more strongly related to valuing forests for its utility in terms of provision of resources than in terms of provision of other, non-material, benefits.

Finally, we have considered that the extraction and use of a forest resource (bushmeat), the frequency of contact with forests and forest cover remaining in the close surroundings is associated with, and thus might be shaping, the way people value forests. However, as in any observational and transversal study, we cannot distinguish between this and the reverse causality: people valuing forests engage more often in forest related activities, consume bushmeat more often or choose to live near forest remnants. Therefore, it is important that future studies investigate these relationships using longitudinal sampling designs or experimental designs that allows for a strong inference of causality (e.g. studies investigating if individual values change as forest cover decrease over time).

### Implications for conservation initiatives

By exploring the factors associated with the diversity of values people attribute to forests, we show that most rural people in a heterogeneous post-frontier region in the Amazon care about forests. Because individual values towards nature are linked to pro-environmental behaviors and attitudes [6, 7, 8], we suggest there are largely unexplored opportunities for conserving privately owned forest through initiatives involving rural communities. This is particularly important given that only 32% of the Amazon lies within protected areas [77], and roughly 53% of the Brazilian native vegetation occurs within private properties where legal protection has recently been reduced by changes in legislation [78].

We have shown that people value forests for the benefits and services they receive not only in terms of consumable resources, but also in terms of non-material benefits, such as recreation, aesthetic, spiritual or cultural well-being, and satisfaction in knowing forests exist now or for future generations. Despite methodological challenges for measuring, mapping and monetizing cultural ecosystem services [78], our findings add to the growing literature suggesting that non-material benefits, which are shaped by intimate human–nature interactions [79, 80, 81], are perceived as equally valuable, and are thus as important for conservation initiatives as the provision of consumable resources.

Knowing why and how people value forests is strategic to conceive initiatives that can be accepted and hence adopted by local people, increasing the chance of success of such efforts. In this sense, our results suggest that forbidding different kinds of forest use can be counterproductive because it decreases the value people attribute to forests and then the willingness to conserve them. However, individual values towards forests are likely to be influenced by demographic variables, such as gender and origin, which should be carefully taken into account when planning community-based conservation initiatives. For instance, it may be important to consider that different groups of people in the same community may need different strategies, as they value forests in different ways, or that some of these groups are more likely to engage in a particular conservation initiative than others are.

Our study also highlights the possibility of a dangerous reinforcing cycle: deforestation and the loss of contact with forests may be related to the erosion of the non-material value attributed to forests, which may in turn facilitate further deforestation. Thus engaging people in forest conservation initiatives is probably more difficult but also more urgent in more altered landscapes. However, we also found that the harvest and consumption of a widely used resource—bushmeat—are likely to increase the material value attributed to forests, and could motivate people to engage in forest conservation. Given that consumption of bushmeat is widespread even in heavily deforested landscapes [37], the incentive for sustainably using this resource could stimulate the acceptance of conservation initiatives also in altered regions where the non-material value attributed to forests may be eroded. This kind of strategy would clearly require the monitoring of game species and a careful plan of sustainable hunting management, as hunting is often linked to the decline and extinction of game species in the Amazon and elsewhere.

## Supporting Information

S1 Fig PCA biplots showing the variation in the scores of the Likert scales for the consumptive and non-consumptive values attributed to forests. (DOCX)

S2 Fig PCA biplots showing the variation in the scores of the Likert scales for the consumptive value (with five items) and non-consumptive value (with four items) attributed to forests. (DOCX)

S1 Table Model selection results for choosing the best proxy for the predictors of consumptive value attributed to forests. (DOCX)

S2 Table Model selection results for choosing the best proxy for the predictors of non-consumptive value attributed to forests. (DOCX)

S3 Table Results for the principal component analysis of the two Likert scales for the consumptive and non- consumptive values attributed to forest with six items each. (DOCX)

S4 Table Results for the principal component analysis of the two Likert scales for the consumptive (with five items) and non- consumptive (with four items) values attributed to forest. (DOCX)

S1 Text Selection of the proxy measures of each of the predictors that best explained the variation in individual values (material and non-material) attributed to forests. (DOCX)

S2 Text Principal component analysis (PCA) for evaluating the unidimensionality of each of the two Likert scales (material and non-material value). (DOCX)
