# Supplementary material for: Who Cares about Forests and Why? Individual Values Attributed to Forests in a Post-Frontier Region in Amazonia
Source: PLoS One. 2016 Dec 12;11(12):e0167691. doi: 10.1371/journal.pone.0167691 (PMC5152861; doi:10.1371/journal.pone.0167691)
Supplement: S1 Fig — (DOCX) [file pone.0167691.s001.docx]

**
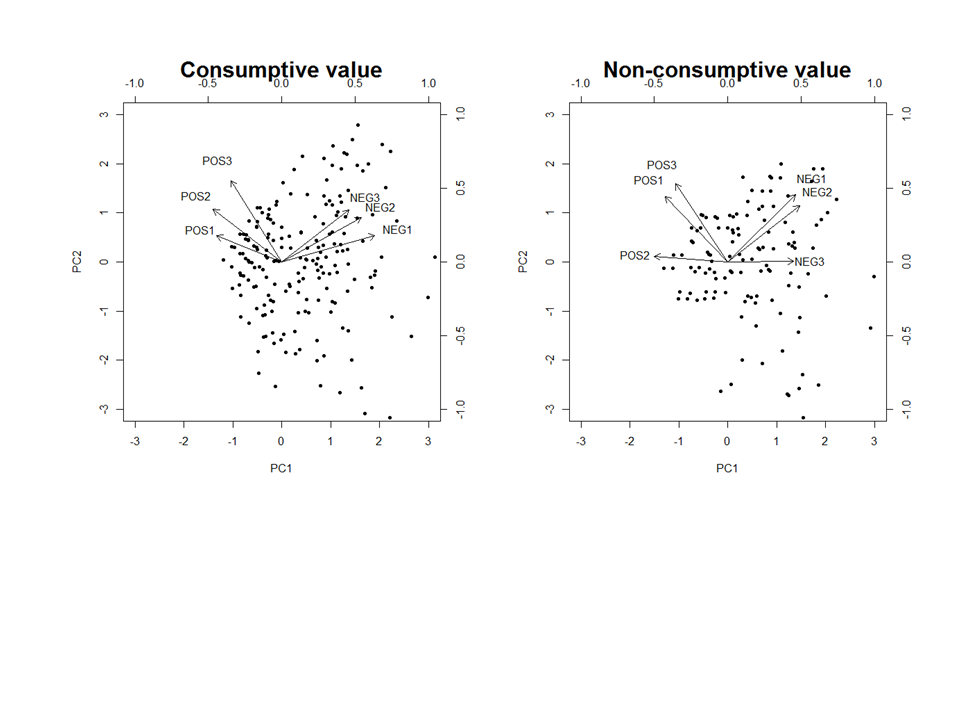
**

**S1 Fig. PCA biplots showing the variation in the scores of the Likert scales for the consumptive and non-consumptive values attributed to forests.** Residents are shown as dots and the six items (three positive – POS and three negative – NEG) as vectors.
