# Supplementary material for: Who Cares about Forests and Why? Individual Values Attributed to Forests in a Post-Frontier Region in Amazonia
Source: PLoS One. 2016 Dec 12;11(12):e0167691. doi: 10.1371/journal.pone.0167691 (PMC5152861; doi:10.1371/journal.pone.0167691)
Supplement: S1 Table — (DOCX) [file pone.0167691.s003.docx]

**S1 Table. Model selection results for choosing the best proxy for the predictors of consumptive value attributed to forests.**

|  | Model | logLik | AICc | wAICc |
| --- | --- | --- | --- | --- |
| Bushmeat consumption | Total number of meals containing bushmeat | -1061.2 | 2132.6 | 0.55 |
|  | Total quantity of consumed bushmeat | -1061.9 | 2133.9 | 0.29 |
|  | Quantity of consumed bushmeat per adult equivalent | -1062.5 | 2135.1 | 0.16 |
| Hunting | Whether any household member had gone hunting | -1060 | 2130.2 | 0.57 |
|  | Whether any household member had gone hunting and succeeded | -1060.6 | 2131.4 | 0.3 |
|  | Total number of hunting events | -1062.2 | 2134.6 | 0.06 |
|  | Total hunting offtake | -1062.3 | 2134.8 | 0.06 |
