# Supplementary material for: Who Cares about Forests and Why? Individual Values Attributed to Forests in a Post-Frontier Region in Amazonia
Source: PLoS One. 2016 Dec 12;11(12):e0167691. doi: 10.1371/journal.pone.0167691 (PMC5152861; doi:10.1371/journal.pone.0167691)
Supplement: S2 Table — (DOCX) [file pone.0167691.s004.docx]

**S2 Table. Model selection results for choosing the best proxy for the predictors of non-consumptive value attributed to forests.**

|  | Model | logLik | AICc | wAICc |
| --- | --- | --- | --- | --- |
| Forest cover | All forest types at 1-km | -693.9 | 1395.8 | 0.28 |
|  | Non-degraded primary forest at 1-km | -694.3 | 1396.6 | 0.18 |
|  | All forest types at 2-km | -694.6 | 1397.3 | 0.14 |
|  | Degraded and non-degraded primary forest at 1-km | -694.6 | 1397.3 | 0.13 |
|  | Degraded and non-degraded primary forest at 2-km | -695.4 | 1399 | 0.06 |
|  | Non-degraded primary forest at 2-km | -695.6 | 1399.3 | 0.05 |
|  | All forest types at 3-km | -695.8 | 1399.8 | 0.04 |
|  | Non-degraded primary forest at 3-km | -696.4 | 1400.9 | 0.02 |
|  | Degraded and non-degraded primary forest at 3-km | -696.4 | 1401 | 0.02 |
|  | All forest types at 4-km | -696.5 | 1401.2 | 0.02 |
|  | All forest types at 5-km | -696.8 | 1401.6 | 0.02 |
|  | Non-degraded primary forest at 4-km | -696.8 | 1401.8 | 0.01 |
|  | Degraded and non-degraded primary forest at 4-km | -696.8 | 1401.8 | 0.01 |
|  | Degraded and non-degraded primary forest at 5-km | -696.9 | 1401.9 | 0.01 |
|  | Non-degraded primary forest at 5-km | -696.9 | 1401.9 | 0.01 |
| Visits to forests | Whether the respondent had visited the forest | -690.9 | 1390 | 0.991 |
|  | Whether the respondent had gone hunting in the forest | -696.2 | 1400.7 | 0.005 |
|  | Total time spent in the forest | -697.3 | 1402.7 | 0.002 |
|  | Total number of visits to forest | -697.6 | 1403.4 | 0.001 |
|  | Total time spent hunting in the forest | -698.4 | 1405.1 | <0.001 |
|  | Total number of hunting events in the forest | -698.7 | 1405.5 | <0.001 |
