# Supplementary material for: Who Cares about Forests and Why? Individual Values Attributed to Forests in a Post-Frontier Region in Amazonia
Source: PLoS One. 2016 Dec 12;11(12):e0167691. doi: 10.1371/journal.pone.0167691 (PMC5152861; doi:10.1371/journal.pone.0167691)
Supplement: S3 Table — (DOCX) [file pone.0167691.s005.docx]

**S3 Table. Results for the principal component analysis of the two Likert scales for the consumptive and non- consumptive values attributed to forest with six items each.**

|  | Factor | Eigenvalue | Proportion explained | Cumulative proportion |
| --- | --- | --- | --- | --- |
| Consumptive  value | 1 | 2.22 | 0.37 | 0.37 |
|  | 2 | 1.13 | 0.19 | 0.56 |
|  | 3 | 0.82 | 0.14 | 0.70 |
|  | 4 | 0.71 | 0.12 | 0.82 |
|  | 5 | 0.63 | 0.11 | 0.92 |
|  | 6 | 0.48 | 0.08 | 1.00 |
| Non-consumptive value | 1 | 1.90 | 0.32 | 0.32 |
|  | 2 | 1.31 | 0.22 | 0.54 |
|  | 3 | 0.87 | 0.14 | 0.68 |
|  | 4 | 0.73 | 0.12 | 0.80 |
|  | 5 | 0.63 | 0.11 | 0.91 |
|  | 6 | 0.55 | 0.09 | 1.00 |
