# Supplementary material for: Who Cares about Forests and Why? Individual Values Attributed to Forests in a Post-Frontier Region in Amazonia
Source: PLoS One. 2016 Dec 12;11(12):e0167691. doi: 10.1371/journal.pone.0167691 (PMC5152861; doi:10.1371/journal.pone.0167691)
Supplement: S4 Table — (DOCX) [file pone.0167691.s006.docx]

**S4 Table. Results for the principal component analysis of the two Likert scales for the consumptive (with five items) and non- consumptive (with four items) values attributed to forest.**

|  | Factor | Eigenvalue | Proportion explained | Cumulative proportion |
| --- | --- | --- | --- | --- |
| Consumptive value | 1 | 2.11 | 0.42 | 0.42 |
|  | 2 | 0.94 | 0.19 | 0.61 |
|  | 3 | 0.79 | 0.16 | 0.77 |
|  | 4 | 0.69 | 0.14 | 0.90 |
|  | 5 | 0.48 | 0.10 | 1.00 |
| Non-consumptive value | 1 | 1.73 | 0.43 | 0.43 |
|  | 2 | 0.87 | 0.22 | 0.65 |
|  | 3 | 0.84 | 0.21 | 0.86 |
|  | 4 | 0.56 | 0.14 | 1.00 |
