# Supplementary material for: Who Cares about Forests and Why? Individual Values Attributed to Forests in a Post-Frontier Region in Amazonia
Source: PLoS One. 2016 Dec 12;11(12):e0167691. doi: 10.1371/journal.pone.0167691 (PMC5152861; doi:10.1371/journal.pone.0167691)
Supplement: S1 Text — (DOCX) [file pone.0167691.s007.docx]

**S1 Text**

We selected the proxy or measure of each of the predictors that best explained the variation in the Likert scale for each type of value attributed to forests with a model selection approach using Akaike’s Information Criterion modified for small samples (AICc) (S1 Table, S2 Table). For each dependent variable (Likert scale of consumptive and non-consumptive values attributed to forests) and each predictor, we compared a candidate set of simple models each containing a different proxy as a fixed factor, and selected the one contained in the top model. To account for the hierarchical nature of the sampling design, we used generalized linear mixed-effects models (GLMM). In all models, landscape was considered a random factor. For the consumptive value attributed to forests, models also included household as a random factor because in most households both household heads were interviewed. As visits to forests were included in the interview only for the household head that more often performed forest activities, all respondents included in the analyses for the non-consumptive value attributed to forests were from distinct households (household was not included as a random factor in this case), and the number of respondents was lower (239 respondents instead of 363). We modeled the dependent variables as a variable with normal distribution and used a linear function (link identity), since Likert scale, which should be constructed summing the values of several items, are not considered ordinal scales, but rather produce interval data [1]. Fixed factors that were continuous variables were standardized so that each had a mean of zero and a standard deviation of one, a procedure adopted to help improving convergence of the fitting algorithm [2].

**References**

1. Carifio J, Perla RJ. Ten common misunderstandings, misconceptions, persistent myths and urban legends about Likert scales and Likert response formats and their antidotes. J Soc Sci. 2007, 3: 106-116.
2. Zuur AF, Ieno EN, Wlaker NJ, Saveliev AA, Smith GM. Mixed effects models and extensions in ecology with R. New York: Springer Science and Business Media; 2009.
