# Supplementary material for: Who Cares about Forests and Why? Individual Values Attributed to Forests in a Post-Frontier Region in Amazonia
Source: PLoS One. 2016 Dec 12;11(12):e0167691. doi: 10.1371/journal.pone.0167691 (PMC5152861; doi:10.1371/journal.pone.0167691)
Supplement: S2 Text — (DOCX) [file pone.0167691.s008.docx]

**S2 Text**

We evaluate the unidimensionality of each of the two Likert scales (one for each type of value) conducting principal component analysis (PCA). We conduct PCA twice for each type of value, first with all six items (S3 Table, S1 Fig) and then excluding those that were less correlated with the other items. For the consumptive value scale we excluded one positive item and for the non-consumptive value scale we excluded two positive items (S4 Table, S2 Fig).
